# Pregnant Women Chronically Infected by *Toxoplasma gondii* with Depressive Disorder: Differential Modulation of Pro-Inflammatory and Anti-Inflammatory Cytokines

**DOI:** 10.3390/pathogens14040330

**Published:** 2025-03-30

**Authors:** Carolina Salomão Lopes, Ricardo José Victal Carvalho, Tamires Lopes da Silva, Heber Leão Silva Barros, Lucas Vasconcelos Soares Costa, Danielly Christine Adriani Maia Mota, Bellisa Freitas Barbosa, Luan Souza Vieira, Talyene Marques de Araújo, Alírio Resende Costa, Ruth Opeyemi Awoyinka, Tiago Wilson Patriarca Mineo, Angélica Lemos Debs Diniz, José Roberto Mineo

**Affiliations:** 1 Laboratory of Immunoparasitology, Department of Immunology, Institute of Biomedical Sciences, Federal University of Uberlândia, Uberlândia 380405-317, MG, Brazil; carolina.lopes@umassmed.edu (C.S.L.); professorricardo@ufu.br (R.J.V.C.); tlopes_s@yahoo.com.br (T.L.d.S.); heberleaosilva@gmail.com (H.L.S.B.); vasconcelos.sc@hotmail.com (L.V.S.C.); ruthawoyinka@gmail.com (R.O.A.); tiago.mineo@ufu.br (T.W.P.M.); 2 Clinical Department, Faculty of Medicine, Federal University of Uberlândia, Uberlândia 380405-317, MG, Brazil; lusovie@gmail.com (L.S.V.); talyene.m.a@hotmail.com (T.M.d.A.); alirioresende@outlook.com (A.R.C.); 3 Laboratory of Immunophysiology of Reproduction, Institute of Biomedical Sciences, Federal University of Uberlândia, Uberlândia 380405-317, MG, Brazil; bellisafb@ufu.br; 4 Gynecology and Obstetrics Department, Faculty of Medicine, Federal University of Uberlândia, Uberlândia 380405-317, MG, Brazil; angelicadiniz@ufu.br

**Keywords:** *Toxoplasma gondii*, chronic infection, pregnancy, depressive disorder, antibodies, cytokines, immune modulation

## Abstract

Depressive disorder during pregnancy is a common condition, affecting approximately 10–15% of pregnant women, and is associated with adverse pregnancy outcomes such as inadequate prenatal care, substance abuse, and fetal growth restriction. Beyond neurotransmitter disturbances, increasing evidence suggests that infectious agents may play a role in the pathophysiology of depression through immune system modulation. *Toxoplasma gondii* infection has been linked to various mental disorders in the general population, including depression and anxiety. This study aimed to investigate whether depressive disorder during pregnancy is associated with chronic *T. gondii* infection by analyzing cytokine levels involved in inflammatory response modulation. Serum levels of TNF, IFN-γ, TGF-β1, IL-6, IL-8, IL-10, and MIF were measured in 79 pregnant women (18–40 years old) during the third trimester of an uncomplicated pregnancy. Participants were divided into four groups: Group I—depressive disorder and *T. gondii* seropositive (n = 19); Group II—no depressive disorder and *T. gondii* seropositive (n = 20); Group III—depressive disorder and *T. gondii* seronegative (n = 20); and Group IV—no depressive disorder and *T. gondii* seronegative (n = 20). Depressive symptoms were assessed using the Edinburgh Postnatal Depression Scale (EPDS) during routine prenatal visits, and blood samples were collected during standard prenatal examinations. Significant differences in cytokine levels were observed among the study groups. Notably, the group with both depressive disorder and chronic *T. gondii* infection exhibited a distinct cytokine profile characterized by significantly elevated TNF, IL-6, and IL-10 levels and significantly reduced IL-8 and MIF levels compared to the other groups. These findings suggest that pregnant women with depressive disorder and chronic *T. gondii* infection exhibit an altered balance of pro- and anti-inflammatory cytokines. This is the first study to investigate the association between serum cytokine levels, depressive disorder, and chronic *T. gondii* infection in pregnant women. Further research is needed to evaluate the potential of these immunobiomarkers as diagnostic tools or for monitoring therapeutic and prognostic strategies in this context.

## 1. Introduction

Depression, a mood disorder, has been considered by the World Health Organization to be one of the most common causes of disability and a public health problem [1]. During pregnancy and the postpartum period, cases of depression and anxiety are frequent, and negligence in the treatment of psychiatric illness has been associated with adverse effects in mothers and babies [2]. The estimated prevalence of depressive disorder (DD) in pregnancy ranges from 10 to 15% in high-income countries [3]; however, in developing countries such as Brazil, the average rates range from 10% to 25% [4,5,6], and it is associated with inadequate prenatal care, drug abuse, low birth weight, fetal growth restriction, preterm labor, risk of preeclampsia, and operative delivery [7].

*Toxoplasma gondii* is an obligate intracellular protozoan that is distributed worldwide. Most infections by *T. gondii* are considered asymptomatic; however, infection by this parasite has been linked to several mental illnesses, including schizophrenia [8,9], anxiety [10], depression [11,12,13], and cognitive impairment [14,15,16,17]. The association between *T. gondii* infection and mood disorders remains controversial. A study conducted in Durango City, Mexico, found no serological evidence of an association between *T. gondii* infection and depression in pregnant women [11]; however, a study carried out in the U.S. reported a link between prenatal depression and anxiety in *T. gondii*-positive women [10].

The pathophysiological mechanism by which *T. gondii triggers* mood disorders is not yet elucidated [13]. It is suggested that in psychosis, the potential mechanism involved in behavioral change may be the direct effect of the parasite on neuronal function and dopamine and serotonin synthesis [18]. Another mechanism is related to the side effects of the immune response against the parasite. Indeed, cytokine variations and mood disorders have been reported [19,20].

During acute *T. gondii* infection, the host immune response produces pro-inflammatory cytokines such as IL-6, TNF, IFN-γ, macrophage migration inhibitory factor (MIF), and IL-8, which help block parasite growth by inducing oxidative stress [21,22,23]. Interestingly, an imbalance in cytokines such as IL-6, TNF, IFN-γ, MIF, IL-17, TGF-β1, and IL-8 [19,24,25,26] are correlated to psychiatric disorders. In the context of mood disorders and infectious disease, the immune response to *T. gondii* has been suggested to contribute to depression through serotonin modulation [13]. It has been reported that during *T. gondii* infection, the production of pro-inflammatory cytokines, such as IL-6 and TNF, and the consequent activation of T CD4^+^ helper cells result in IFN-γ secretion, inducing the activation of IDO (indoleamine 2,3-dioxygenase), which depletes tryptophan, ultimately reducing serotonin levels in the brain.

Considering previous findings reported in the literature, the present work aimed to evaluate the cytokine profile associated with depressive disorder in *T. gondii*-seropositive pregnant women.

## 2. Methods and Materials

### 2.1. Participants

A cross-sectional study was conducted in 2016–2018 with pregnant women at Clinical Hospital of the Federal University of Uberlândia. This study was approved by the Committee of Ethics in Research from Federal University of Uberlândia (CEP-UFU), protocol # CAAE 55483716.2.0000.5152, on 10 May 2016, and all patients agreed to be participants and signed a consent form. Pregnant women during the third trimester were enrolled during routine consultation. Each participant completed a previously designed questionnaire consisting of short questions regarding demographic data such as age, average household income, occupational status, educational background, previous history of pregnancy, medication intake, relationship status (e.g., married or unmarried), and gestational age at the time of the interview (weeks). All questionnaires were evaluated carefully, and the following inclusion/exclusion criteria were used: patients should be between 18 and 41 years old; have no records of serious obstetric, infectious, and psychiatric diseases; and not be suspected to have congenital toxoplasmosis.

Depression was diagnosed using the EPDS scale (Edinburgh Postnatal Depressive Scale) (Cox et al., 1987 [27]; Santos et al., 2007 [28]). This test consists of 10 self-rating questions that were designed and validated for depression in postpartum women, and it was validated for depression during pregnancy (Castro-E-Couto et al., 2015 [29]). Patients were diagnosed with depression when EPDS scores were ≥11.

### 2.2. Study Design

The power and sample size were calculated. The sample size calculation was based on an expected prevalence of depression in pregnant woman of 15% (p), a 95% confidence interval (Z = 1.95), and a 5% desired absolute precision (d) [n = p(1 − p)Z^2^/d^2^], determining a sample of 194 individuals, according to Bakker et al., 2020 [30]. A total of 200 patients were included in this study, and their medical records were carefully reviewed to verify information regarding IgG anti-*T. gondii* and other comorbidities. However, only 79 patients met the criteria of not being under medication for depression or any other inflammatory comorbidity and/or agreed to participate in the study. The patients were divided into four groups according to the EPDS and serological screening for *T. gondii* IgG: Group I (IgG+/DD+)—pregnant women with depressive disorder (EPDS ≥ 11 points) and seropositive for *T. gondii* (n = 19); Group II (IgG+/DD−)—pregnant women without depressive disorder (EPDS < 11 points) and seropositive for *T. gondii* (n = 20); Group III (IgG−/DD+)—pregnant women with depressive disorder (EPDS ≥ 11 points) and seronegative to *T. gondii* (n = 20); and Group IV (IgG−/DD−)—pregnant women without depressive disorder EPDS < 11 points) and seronegative to *T. gondii* (n = 20). There were 19 patients in Group I; in this sense, we normalized all groups with 20 patients each. To validate our new sample size (n = 79), the power size of the sample was calculated according to Bakker et al., 2020 [30]. Assuming a 5% error, it was found to be 99.5%.

### 2.3. Assessment of T. gondii Seropositivity

Patients’ serology was confirmed by indirect ELISA, as described by Silva et al. (2002) [31], with modifications. Briefly, high-binding microtiter plates (Costar-Corning Incorporated) were coated with 10 μg/mL of STAg (soluble *Toxoplasma* antigen) diluted in 0.06 M carbonate buffer (pH 9.6) and incubated overnight at 4 °C. Then, plates were blocked with 5% skim fat milk in PBS-T for 1 h at room temperature. After that, serum samples were diluted in 1% skim fat milk diluted in PBS-T at 1:64 and placed into plate wells in duplicate and incubated for 1 h at 37 °C. In sequence, an incubation with peroxidase-labeled goat anti-human IgG (1:2000, Sigma-Aldrich Chemical Co., St. Louis, MO, USA) was performed for 1 h at 37 °C. Finally, the enzyme substrate (0.03% H_2_O_2_ and 0.01 M ABTS) was added for revealing enzymatic activity. Plates were washed with PBS-T between each step described before. The OD values were determined at 405 nm. Results were expressed as ELISA index (EI), according to the following formula: EI = OD sample/OD cutoff. Samples with EI values ≥ 1.2 were considered positive.

### 2.4. Cytokine Measurements

Blood samples were obtained from remnants of serum samples from prenatal routine tests. To avoid variations due to circadian rhythm, all appointments for blood collection were scheduled in the morning, between 7:00 and 8:00 a.m. After collection, the serum samples were stored at −70 °C until cytokine analysis. The IL-6, TNF, IFN-γ, IL-8, IL-10, TGF-β1, and MIF cytokines were measured by sandwich ELISA according to the manufacturer’s (BD Bioscience or R&D Systems, San Jose, CA, USA and Minneapolis, MN, USA, respectively) instructions in each cytokine kit. The data were expressed in pg/mL according to a standard curve of each cytokine.

### 2.5. Statistical Analysis

The data analysis was conducted using GraphPad Prism software, version 6.0 (GraphPad Software, Inc., San Diego, CA, USA). A bivariate analysis was first performed to select variables for the subsequent multivariate analysis. The association between depression, anti-*T. gondii* IgG, and the cytokine profile in pregnant women were evaluated using the Brunner–Munzel test, calculated in R, to account for differences between groups. The Brunner–Munzel test was used to compare cytokine levels between two groups, while the Kruskal–Wallis test was applied for multiple group comparisons, followed by Dunn’s post hoc test to identify specific group differences. Dunn’s test, followed by Holm’s method, was chosen for its suitability for non-parametric data and its ability to control Type I error, making it ideal for our dataset, which did not meet the assumptions of normality. Demographic data were evaluated using the chi-square test. Statistical significance was defined as *p* < 0.05.

To further explore the underlying patterns in the data while summarizing major sources of variation, Principal Component Analysis (PCA) was performed, and the first six principal components (PCs) were evaluated. PCA was applied to cytokine levels, ELISA indexes, and EPDS scores to reduce the dimensionality of the dataset and capture the most significant variance components.

## 3. Results

### 3.1. Pregnant Women Present Significant Differences Among EPDS Scores from Experimental Versus Control Groups Without Previous Occurrence of Depression

As shown in Table 1, pregnant women from groups I and III presented EPDS scores significantly higher than those from groups II and IV (*p* < 0.001), and the number of these patients without previous depression occurrence was higher than that with previous depression episode (*p* < 0.05). When the duration of pregnancy among participants was compared with the scores on the EPDS, no significant differences were observed (*p* > 0.05). The age means of the pregnant women presenting seropositivity for *T. gondii* antibodies were higher than those seronegative (*p* < 0.05). Related to marital status, the number of pregnant women living with their partners was higher than other statuses (*p* < 0.05). Regarding previous pregnancies, most participants were multiparous (*p* < 0.001) and predominantly had an education level of elementary or high school (*p* < 0.01).

### 3.2. ELISA Indexes for IgG Antibodies to T. gondii and EPDS Scores Are Validated for Groups I, II, III, and IV of Patients, but No Correlation Was Found Between Both Parameters

**Figure 1:**
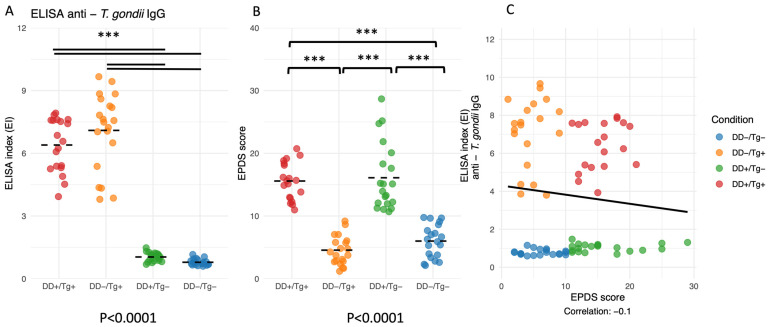
(**A**) Detection of IgG antibodies against *T. gondii* in patient Groups I-IV using ELISA, with the results expressed as ELISA index values, which quantify relative antibody concentration by comparing the optical density (OD) of each sample to a cutoff value derived from control samples. (**B**) Determination of the Edinburgh Postnatal Depressive Scale (EPDS) scores for Groups I–IV of patients. (**A**,**B**) The mean for each group is indicated by a horizontal line in the plot, representing the average value of the data points within that group. (**C**) Correlation between ELISA index and EPDS scores for Groups I–IV of patients. *** *p* < 0.001.

When determining the levels of IgG antibodies to *T. gondii*, it was possible to observe that the ELISA indexes for Groups I and II were statistically higher than those observed for Groups III and IV, confirming that the former groups were from patients infected by this parasite, whereas the latter two groups were IgG-negative (*p* < 0.0001) (Figure 1A). Also, the EPDS scores for both groups of depressive patients (Group I and Group III) were statistically higher when compared to both groups of non-depressed patients (Group II and Group IV) (*p* < 0.0001) (Figure 1B). However, when a comparison was made between ELISA indexes and EPDS scores, no correlation was observed between the two parameters (*p* > 0.05) (Figure 1C).

### 3.3. Higher Serum Levels of IL-10 and IL-6, but Lower Levels of IL-8, Cytokines Are Associated with Patients Chronically Infected by T. gondii

**Figure 2:**
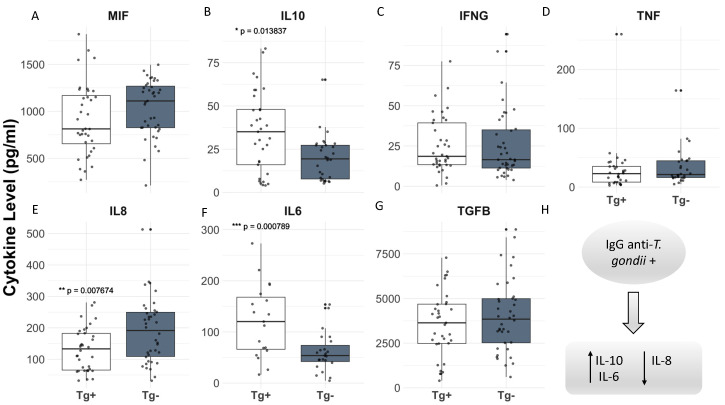
(**A**–**H**) Serum levels of pro-inflammatory and anti-inflammatory cytokines (MIF, IFNg, TNF, IL8, figures A, C, D and E, respectively; IL10, IL6, TGFB, figures B, F and G, respectively) were measured and compared between chronically *T. gondii*-infected patients (Groups I and II) and uninfected controls (Groups III and IV). Statistical significance was assessed using the Brunner–Munzel test, with significant differences (*p* < 0.05) displayed in the graph, indicating the decreasing corresponding *p*-values, as follows: * *p* = 0.013837; ** *p* = 0.007674; and *** *p* = 0.000789.

When analyzing the cytokine levels in the groups of patients with seropositivity for *T. gondii* (Groups I and II), compared to seronegative patients (Groups III and IV), no significant changes were observed in the levels of MIF, IFN-γ, TNF, and TGF-β1 associated with the positivity to *T. gondii* for the chronically infected patients (Figure 2A,C,D,G). However, higher levels of IL-10 and IL-6 (Figure 2B,F) and lower levels of IL-8 (Figure 2E) were found in the serum levels of patients infected by this parasite.

### 3.4. Higher Serum Levels of IL-10, TNF and TGF-β1, but Lower Levels of IL-8, Cytokines Are Associated with Patients Presenting Depressive Disorder

**Figure 3:**
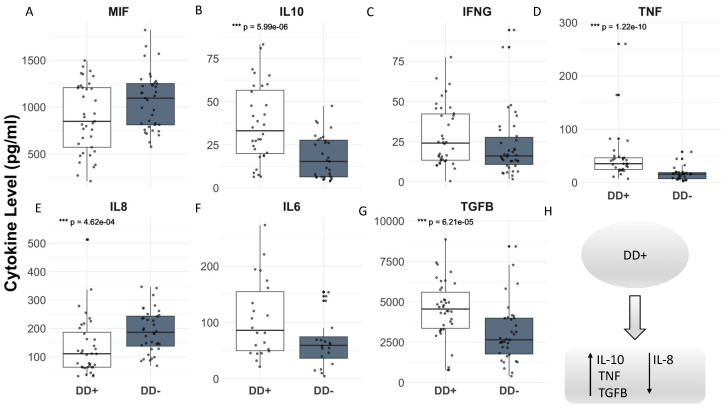
(**A**–**H**) Serum levels of pro-inflammatory and anti-inflammatory cytokines (MIF, IFNg, TNF, IL8, figures A, C, D and E, respectively; IL10, IL6, TGFB, figures B, F and G, respectively) were measured and compared between groups with depressive disorder (Groups I and III) and groups without this mental problem (Groups II and IV). Statistical significance was assessed using the Brunner–Munzel test, with significant differences (*p* < 0.05) displayed in the graph, indicating the range of the corresponding *p*-values, as follows: *** *p* = 4.62 × 10^−4^; *** *p* = 6.21 × 10^−5^; *** *p* = 5.99 × 10^−6^; and *** *p* = 1.22 × 10^−10^.

When analyzing the cytokine levels in the groups of patients with depressive disorder (Groups I and III), as determined by EPDS scores ≥ 11, in comparison with the groups without this disorder (Groups II and IV), there were no observed changes in the levels of MIF, IFN-γ, and IL-6 associated with this mental disorder (Figure 3A,C,F). In contrast, higher levels of IL-10, TNF, and TGF-β1 (Figure 3B,D,G) and lower levels of IL-8 (Figure 2E) were found in the serum levels of these groups of patients.

### 3.5. The Cytokine Level of MIF Is the Lowest for the Patients Presenting the Association of Infection by T. gondii and Depressive Disorder When Compared with All Other Groups of Patients

**Figure 4:**
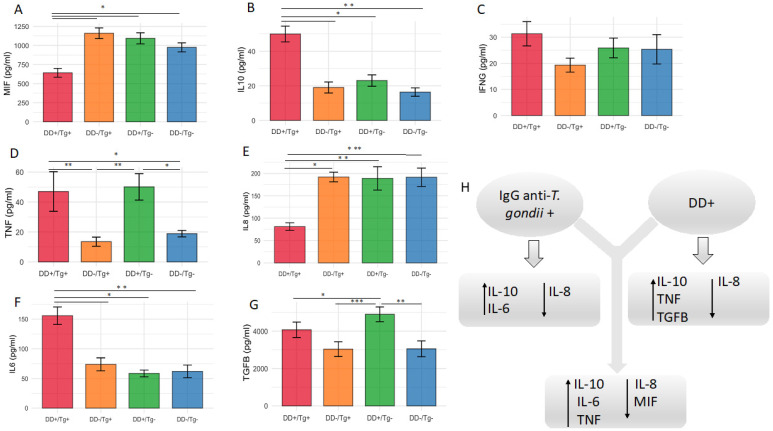
(**A**–**H**) Determination of the levels of pro-inflammatory and anti-inflammatory cytokines (MIF, IFNg, TNF, IL8, figures A, C, D and E, respectively; IL10, IL6, TGFB, figures B, F and G, respectively) in serum samples of patients presenting infection by *T. gondii* and depressive disorder (Group I) compared with all groups of patients (Groups II, III, and IV) The Kruskal–Wallis test was used for multiple groups comparisons, followed by Dunn’s post hoc test to identify specific group differences. Holm’s method was applied to reduce the risk of false positives * *p* < 0.05; ** *p* < 0.01; *** *p* < 0.001.

When evaluating cytokine levels considering the patients presenting the association of both clinical situations, i.e., infection by *T. gondii* and depressive disorder (Group I), compared with all remaining groups of patients (Groups II, III and IV), it was found that the mean level of MIF for these patients was the lowest one (Figure 4A) (*p* < 0.05). This group of patients also showed higher levels of IL-10, TNF, and IL-6 (Figure 4B,D,F) (*p* < 0.05; *p* < 0.01), associated with lower levels of IL-8 (Figure 4E) (*p* < 0.05; *p* < 0.01; *p* < 0.001).

**Figure 5:**
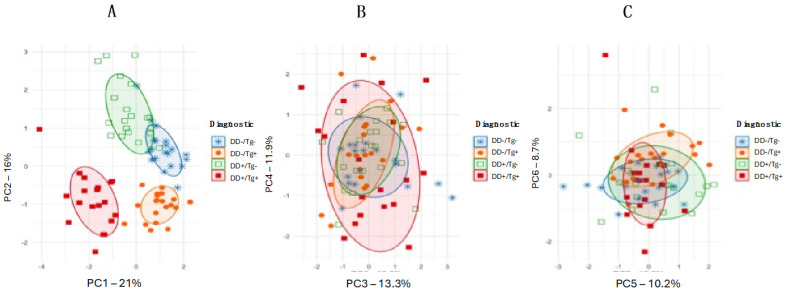
Principal Component Analysis of cytokine levels, ELISA indexes, and EPDS scores among the four diagnostic groups. The panels (**A**–**C**) show different combinations of principal components (PC1 vs. PC2, PC3 vs. PC4, and PC5 vs. PC6) to demonstrate variance accounts for each component. Colored ellipses indicate the 95% confidence intervals for each diagnostic group: DD−/Tg− (blue stars), DD−/Tg+ (orange circles), DD+/Tg− (green squares), and DD+/Tg+ (red squares).

To account for potential confounding factors and reduce noise, Principal Component Analysis (PCA) was performed to provide a lower-dimensional representation of the dataset. The analysis included cytokine levels, ELISA indexes, and EPDS scores to identify the primary sources of variance and elucidate the relationships between biological and psychological markers. As shown in Figure 5A–C, the first two principal components (PC1 and PC2) explained 21% and 16% of the total variance, respectively, with subsequent components contributing minimally. The clear separation of the DD+/Tg+ and DD−/Tg+ groups along PC1 and PC2 demonstrates the discriminatory power of PCA in distinguishing these groups and reinforces the findings presented in Figure 4.

## 4. Discussion

*Toxoplasma gondii* is a protozoan parasite that has the ability to invade the brain and can induce significant behavioral changes. The current literature presents substantial evidence of an association between *T. gondii* infection and various psychiatric disorders, including depressive disorder. However, additional studies are needed to confirm this association by examining both the seroepidemiology of *T. gondii* infection in patients with these disorders and the biological markers involved, particularly those linked to inflammatory damage in the brain.

The main aim of the present study was to assess the profiles of pro-inflammatory and anti-inflammatory cytokines associated with depressive disorder in *T. gondii*-seropositive pregnant women.

One of the most accepted current theories about mental disorders is their correlation with neurotransmitters, like dopamine, serotonin, and noradrenaline. However, many patients do not respond well to current antidepressant medications, likely due to other factors that may contribute to this lack of effectiveness. One of these suggests that *T. gondii* infection may be linked to certain mental disorders by directly impairing neuronal function in areas where parasite cysts are present. This could lead to apoptosis, changes in neuronal protein expression, or alterations in dopamine production. Alternatively, the presence of *T. gondii* in the brain might indirectly disrupt neuroplasticity, leading to neurodegeneration and neurotransmitter imbalances [32]. Thus, the neuropsychiatric disorders may be a result of *T. gondii* infection because of the tissue cyst location in the brain and the damage induced by host immune responses to the parasite [33,34].

Another potential mechanism is the effect of *T. gondii* infection on endocrine pathways, such as the upregulation of thyroid peroxidase autoantibodies (TPOs) in pregnant women [35]. It has been described that women who are TPO-positive during the postpartum period are more susceptible to depression [36]. Also, there are interesting findings related to two genes encoding tyrosine and phenylalanine hydroxylases due to *T. gondii* infection that led to the production of L-DOPA, a precursor of dopamine, and may directly affect the behavioral changes in infected hosts [33,37,38]. However, there are some disagreements related to the data described in the literature concerning this issue, as some investigations did not find an association of *T. gondii* IgG seropositivity with depressive disorder. In this context, Sugden et al. (2016) found that *T. gondii* seropositivity was not significantly associated with major depression in a population-based cohort in New Zealand [39]. Similarly, in a meta-analysis of 50 studies on *T. gondii* infection for major psychiatric disorders versus healthy controls, no association between *T. gondii* IgG seroprevalence and major depression was found [8]. In a case–control study, Alvarado-Esquivel did not find an association between *T. gondii* exposure and depression in pregnant women [40].

It is already known that cytokines are key mediators of the immune response, and they have been extensively studied for decades. In fact, considering that they play different functions, a fine adjustment of their expression and release is necessary; otherwise, instead of protection, they can cause immunopathological processes. Among their properties, some of them have synergistic action, in contrast with others exhibiting a clear antagonistic function. In this context, IFN-γ, TNF-α, and MIF are good examples of pro-inflammatory cytokines that play a key role in promoting inflammation and apoptosis, whereas others, such as IL-10, exhibit a significant anti-inflammatory effect, and insufficient production can lead to severe tissue destruction due to uncontrolled inflammation. In contrast, there are other cytokines, such as IL-6 and TGF-β1, with primary functions showing a dual-role cytokine with both pro- and anti-inflammatory effects depending on the cell type and context where they are released [21,22,23,24,25].

In the present study, our data revealed no association between the levels of seropositivity for IgG antibodies to *T. gondii* and levels of depressive disorder determined by the EPDSs, nor the correlation between levels of IgG and EPDS scores. However, when patients with depressive disorder were compared with those without depression, significant differences were observed in cytokine response, with higher levels of IL-10, TNF, and TGF-β1 and lower levels of IL-8 for depressive patients. Compared to pregnant women seronegative to *T. gondii*, pregnant women with positive serology showed higher levels of IL-10 and IL-6 and lower levels of IL-8. In addition, when cytokine levels from all groups were compared, the group of patients with both clinical situations, i.e., depressive disorder and *T. gondii* infection, was the only one that presented specifically lower levels of MIF, combined with higher levels of IL-10, IL-6, and TNF and lower levels of IL-8.

During *T. gondii* infection, the immune system balances pathogen control and tissue protection through a coordinated cytokine response. IL-6, TNF-α, and IL-8 drive early inflammation, with IL-8 recruiting neutrophils to enhance immune defense. IFN-γ, produced by T-helper 1 and natural killer cells, activates macrophages and antimicrobial mechanisms essential for limiting parasite replication. MIF, released by macrophages, amplifies inflammation by promoting TNF-α, IL-6, and IL-12 secretion. Interestingly, *T. gondii* expresses its own MIF homolog, modulating host immunity to aid parasite survival. However, while these pro-inflammatory responses are essential for controlling the parasite, excessive inflammation can cause tissue damage and pathology [19,24,25,26].

To counteract this, IL-10 and TGF-β1 act as key regulators. IL-10 suppresses pro-inflammatory cytokine production, while TGF-β1 modulates immune activation. This interplay between inflammatory and regulatory cytokines is crucial for maintaining immune homeostasis. A delicate balance between these responses ensures effective control of parasite replication while preventing immunopathology. Disruptions in this equilibrium can either lead to uncontrolled inflammation, exacerbating tissue damage, or insufficient immune activation, allowing parasite persistence [21,22].

In this context, the seropositivity for IgG antibodies to *T. gondii* in pregnant women during the third trimester may reflect an altered cytokine balance. This could indicate (i) a shift towards an anti-inflammatory/inflammatory cytokine profile, contrary to what is typically observed in healthy third-trimester pregnancies, and (ii) lower levels of MIF, in contrast to findings in pregnant women with depressive disorders during the third trimester. The Th1-associated immune profile is responsible for host resistance to *T. gondii* infection even during pregnancy, leading to the secretion of IFN-γ, in addition to other pro-inflammatory cytokines, such as IL-6 and TNF-α [33,41].

The immunologic adaptation of pregnant women changes according to the trimester of pregnancy, from pro-inflammatory in the first trimester to anti-inflammatory in the second and then returning to pro-inflammatory in the third. The release of placental cytokines (IL-1β, TNF-α, LIF/IL-6, and IFN-γ) from immune (Mus, NKs, and DCs) and nonimmune cells, the decidual cells and trophoblasts [42], during the third trimester, may contribute to triggering the changes observed in the hippocampus, prefrontal cortex, and anterior cingulate, as well as in the neuroendocrine activity of the hypothalamus and pituitary and adrenal glands. These features and the processes occurring in the placental tissues could elicit symptoms of depression during pregnancy. The release of pro-inflammatory cytokines enhances the altered neuroendocrine responses in women exhibiting depressive disorder during pregnancy [43].

The role of TNF and IFN-γ in inducing depressive symptoms is significant. These cytokines stimulate the production of indoleamine 2,3-dioxygenase (IDO) and then tryptophan (Trp) depletion along the kynurenine pathway, resulting in reduced 5-hydroxytryptamine (5-HT, serotonin) production in the brain [33,44].

Analyzing the immunologic changes that accompany depressive disorder, one meta-analysis of 24 studies on clinical patients reports significantly higher concentrations of the pro-inflammatory cytokines TNF-α and IL-6 in depressed patients compared with control ones [45]. In Brazil, Miranda et al. found elevated levels of IL-6 in patients with mood disorders [46]. Similarly, current depressive symptoms during pregnancy have been associated with elevated maternal serum IL-6 and TNF-α [47,48], showing an increasing trend across pregnancy and a significant increase at postpartum [47], and the relationship between symptom severity and both IL-6 and TNF-α was equivalent to a small effect size [48]. IL-8 is produced by human uterine cervical tissue and participates in its ripening and dilatation during labor [49]. In pregnant physiologic adaptation, the levels of IL-8 show a U-shaped curve, being high in the first trimester, decreasing in the second, and then returning to high titers at the end of pregnancy [47]. MIF is associated with the pathobiology of depression. Many investigations have identified MIF expression in the brain in significant areas associated with the behavioral symptoms of depression [50]. This cytokine may be involved in interaction with lifestyle, physical exercises, and pharmacological effects in antidepressant treatments, as it could be associated with neurogenesis. Thus, it has been investigated as a biomarker in patients presenting major depressive disorder and other mood disorders when its level is low. However, there are many aspects to be investigated concerning the association between MIF and depression, and future studies should work to clarify the relationship between central and peripheral MIF in depression. Further research is needed to clarify whether MIF acts as a pro-depressant or antidepressant and to define its role in the pathobiology of depression. Analyzing its connections with factors within and beyond the monoamine and neurogenic pathways, both known to influence depression, could provide valuable insights [50]. Also, Musil et al. (2011) reported that elevated MIF levels combined with reduced levels of TGF-β support the importance of the regulatory cytokines in major depressive disorder [51]. Given these findings and the unclear pro-inflammatory role of MIF, along with its emerging associations with perinatal outcomes and psychosocial influences during pregnancy, further studies are warranted, particularly to assess the dynamics of MIF changes over time during pregnancy [52]. Concerning the results of the present study, when evaluating the cytokine levels considering the patients presenting the association of both clinical situations, i.e., infection by *T. gondii* and depressive disorder (Group I), compared with all remaining groups of patients (Groups II, III and IV), it was found that the mean level of MIF for these patients was the lowest one, complemented by higher levels of IL-10, TNF, and IL-6 but lower levels of IL-8.

Even though many studies are highlighting the association between latent toxoplasmosis and mental disorders [53], it is difficult to establish a direct causal effect of toxoplasmosis in the pathogenesis of psychiatric disorders without cause–effect studies. Indeed, the simple presence of *T. gondii* during pregnancy is not definitely the causal factor per se, but it could be because of the profile of immune responses and the hormonal changes, which may result in the severity of the disease by infection of a specific strain type of parasite [33,34,36].

## 5. Limitation

This study has limitations that should be considered. Firstly, the pregnant women who need public health services in this region usually do not have access to preventive health assistance during the beginning of pregnancy. This is why we chose to enroll only the pregnant women in their third trimester in this study. This decision presented a limitation because it was not possible to observe the association between depressive disorder and the chronic phase of *T. gondii* during the whole period of pregnancy. Secondly, the body mass index and/or weight gain during the pregnancy would be relevant information, as these conditions may cause inflammation, which may interfere with cytokine production. Because only pregnant women were enrolled in this work, it was not possible to evaluate both parameters. Therefore, the limitations should be considered when interpreting the results of this study.

## 6. Conclusions

**Figure 6:**
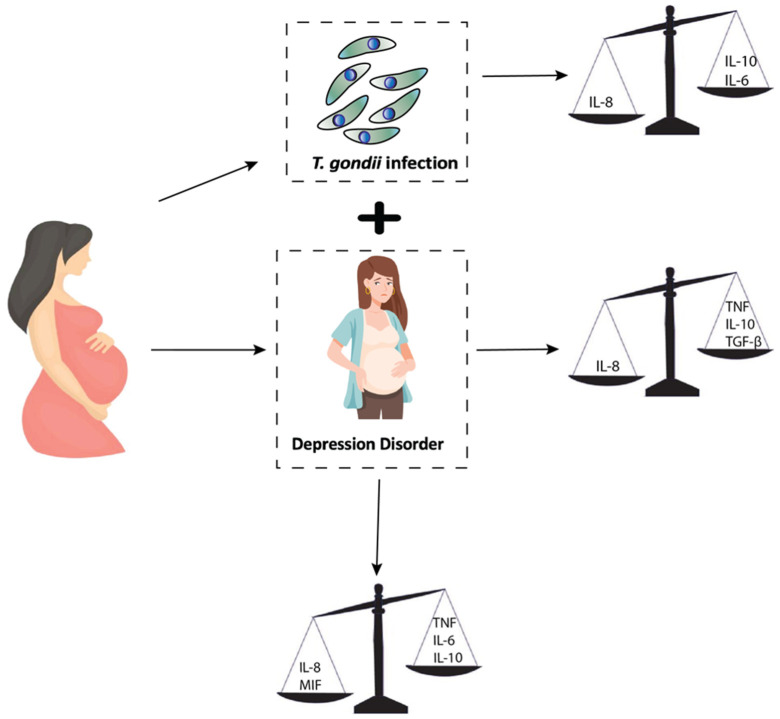
Summary of key findings. Illustration of the interaction between *T. gondii* infection and depressive disorder during pregnancy, highlighting their combined effects on cytokine balance. Chronic phase of *T. gondii* infection is associated with reduced IL-8 and increased IL-10 and IL-6, while depression leads to elevated TNF, IL-10, and TGF-β. When both conditions coexist, the imbalance intensifies, with higher TNF, IL-6, and IL-10 and lower IL-8 and MIF levels. This figure summarizes these cytokine shifts, emphasizing the immune dysregulation linked to both conditions.

Taken together, it can be concluded that pregnant women in the third trimester of pregnancy with depressive disorder and the presence of chronic *T gondii* presented significant changes in the levels of immunomodulatory cytokines, as shown in Figure 6. However, these alterations were not sufficient to exacerbate this psychiatric disease during pregnancy, even considering that these molecules are key components of the immune response.

## Figures and Tables

**Table 1:** Characteristics of the patients from Groups I, II, III, and IV enrolled in the present study.

Characteristics	Group I DD+Tg+ (n = 19)	Group II DD−Tg+ (n = 20)	Group III DD+Tg− (n = 20)	Group IV DD−Tg− (n = 20)	*p*-Value
Maternal age (year) ± SD *	31 ± 6.63 ^a^	30 ± 5.88 ^ab^	27 ± 5.90 ^ab^	26 ± 5.31 ^b^	0.0174
Gestational age (weeks) ± SD	29 ± 8.10	31 ± 8.50	32 ± 7.15	32 ± 7.75	0.1571
EPDS score (score mean) ± SD *	15.6 ± 2.9 ^a^	4.5 ± 2.3 ^b^	16.2 ± 5.5 ^a^	5.8 ± 2.8 ^b^	0.0001
**Marital status**					
Married/living with a partner	14 (74%)	18 (90%)	10 (50%)	12 (60%)	0.0359
Single/separated/divorced/ living without a partner	5 (26%)	2 (10%)	10 (50%)	8 (40%)	
**Degree of education**					
Elementary/High school	9 (47%)	14 (70%)	15 (75%)	19 (95%)	0.0099
Undergraduate	10 (53%)	6 (30%)	5 (25%)	1 (5%)	
**Occupancy**					
Housewife	9 (47%)	7 (35%)	10 (50%)	6 (30%)	0.5387
Daily laborer/employed	10 (53%)	13 (65%)	10 (50%)	14 (70%)	
**Average household income**					
<minimum wage	7 (37%)	9 (45%)	10 (50%)	11 (55%)	0.7326
>minimum wage	12 (63%)	11 (55%)	10 (50%)	9 (45%)	
**Parity**					
Primiparous	7 (37%)	7 (35%)	3 (15%)	15 (75%)	0.0009
Multiparous	12 (63%)	13 (65%)	17 (85%)	5 (25%)	
**Type of previous delivery**					
Cesarean section	5 (42%)	5 (39%)	6 (35%)	3 (60%)	0.8246
Spontaneous labor	7 (58%)	8 (61%)	11 (65%)	2 (40%)	
**Planed Pregnancy**					
Yes	5 (26%)	9 (45%)	5 (25%)	10 (50%)	0.2659
No	14 (74%)	11 (55%)	15 (75%)	10 (50%)	
**Chronic disease**					
Yes	3 (16%)	4 (20%)	5 (25%)	6 (30%)	0.8036
No	16 (84%)	16 (80%)	15 (75%)	14 (70%)	
**Previous depression**					
Yes	2 (10%)	1 (5%)	5 (25%)	0	0.0419
No	17 (90%)	19 (95%)	15 (75%)	20 (100%)	
**Alcohol consumption**					
Yes	1 (5%)	2 (10%)	2 (10%)	2 (10%)	0.9999
No	18 (95%)	18 (90%)	18 (90%)	18 (90%)	
**Smoking**					
Yes	0	2 (10%)	3 (15%)	0	0.1514
No	19 (100%)	18 (90%)	17 (85%)	20 (100%)	
**Negative life events**					
Yes	6 (31%)	3 (15%)	10 (50%)	5 (25%)	0.1094
No	13 (69%)	17 (85%)	10 (50%)	15 (75%)	

*^,a^ or ^b^: same letters, no significant difference; ^a^ and ^b^: different letters, significant differences.

## Data Availability

The original contributions presented in this study are included in the article. Further inquiries can be directed to the corresponding author.
